# Paradox breaker BRAF inhibitors have comparable potency and MAPK pathway reactivation to encorafenib in BRAF mutant colorectal cancer

**DOI:** 10.18632/oncotarget.27681

**Published:** 2020-08-25

**Authors:** Oliver J. Pickles, Aneta Drozd, Louise Tee, Andrew D. Beggs, Gary W. Middleton

**Affiliations:** ^1^Institute of Immunology and Immunotherapy, University of Birmingham, Edgbaston, Birmingham, UK; ^2^Institute of Cancer and Genomic Sciences, University of Birmingham, Edgbaston, Birmingham, UK; ^*^These authors contributed equally to this work

**Keywords:** colorectal cancer, BRAF, paradox breaker, encorafenib, PLX8394

## Abstract

The BEACON CRC trial demonstrated a survival advantage over chemotherapy for a combination of targeted agents comprising the potent BRAF inhibitor encorafenib together with cetuximab and binimetinib. Resistance to BRAF inhibition in CRC arises in part through the generation and activation of RAF dimers resulting in MEK-ERK pathway reactivation. Paradox breaker BRAF inhibitors, such as PLX8394, are designed to inhibit RAF dimer formation. We analyzed whether paradox breakers reduce pathway reactivation and so have enhanced potency compared with encorafenib in BRAF mutant CRC. The potency of encorafenib and PLX8394 was greater than vemurafenib and the degree of pathway reactivation somewhat less. However, dose response curves for encorafenib and PLX8394 were similar and there was no significant differences in degree of pathway reactivation. To our knowledge these data represent the first comparative data of encorafenib and paradox breaker inhibitors in BRAF mutant CRC. Whilst these results support further investigation of PLX8394, all three agents tested reactivated the pathway in melanoma cells, a disease in which monotherapy is effective. Strategies focused on restricting RAF dimerization fail to address the impact that specific context of BRAF mutation in CRC has on targeted therapy outcomes.

## INTRODUCTION

Around 10% of patients with metastatic colorectal cancer (CRC) harbour a BRAF mutation. This confers a significantly worse outcome with chemotherapy, independent of associated clinicopathological features also known to be prognostic [1]. Recently results of the BEACON CRC trial demonstrated an enhanced survival for chemo-refractory BRAF mutant CRC patients for the combination of the BRAF inhibitor encorafenib together with the EGFR inhibitor cetuximab and the MEK inhibitor binimetinib compared with the control group of cetuximab and irinotecan-based therapy. Median overall survival was 9 months in the triplet treated group [2]. The addition of EGFR and MEK inhibition to the BRAF inhibitor backbone is predicated on the activation of RAS observed following BRAF inhibition. Inhibition of BRAF causes downregulation of ERK-induced negative feedback on EGFR-RAS signalling. The resultant activation of RAS drives BRAF/CRAF hetero- and homo-dimerization, which culminates in reactivation of MEK and ERK [3]. Whilst the BEACON CRC results are promising, only a quarter of patients responded to the triple targeted regime. Supplemental biomarkers are likely critical in patient selection for these therapies, however it is also possible that therapeutic efficacy can be increased by optimisation of the various inhibitors in the combination.

Encorafenib is a group 1 BRAF inhibitor that selectively inhibits active BRAF monomers. However, in RAF dimers, binding of the inhibitor to one RAF protomer activates the other, a process known as negative co-operativity. This phenomenon causes pathway reactivation and resistance to BRAF inhibition in BRAF mutant cells and paradoxical pathway activation in BRAF wild-type cells [4–6]. PLX8394 is a paradox-breaker BRAF inhibitor which inhibits BRAF dimerization and which does not result in paradoxical activation in BRAF wild-type cells [7]. It disrupts the RAF dimer interface *via* a strong interaction with the Leucine 505 residue, which is situated close to the αC helix which is critical to dimerization. Additionally, group 1 inhibitors up-regulate EGFR ligands whereas PLX8394 does not. Given that dimerization and enhanced EGFR-RAS signalling drives pathway reactivation, we reasoned that PLX8394 may be a more effective BRAF inhibitor in BRAF mutant CRC for use in combination therapy. Thus, we compared the potency and degree of pathway reactivation of encorafenib and PLX8394 together with a commonly used group 1 inhibitor vemurafenib. Whilst PLX8394 and encorafenib possess different modes of actions, the potency and degree of pathway reactivation of encorafenib and PLX8394 were comparable and both were superior to vemurafenib.

## RESULTS

The differential potency of encorafenib, PLX8394 and vemurafenib was assessed by measuring the effect of each BRAF inhibitor on cell viability using an ATP-based reporter assay (RealTime-Glo™ MT Cell Viability Assay). BRAF V600E-mutated colorectal (WiDr, RKO, Colo 201, and LS411N) and melanoma (G361 and A375) cell lines were treated with each drug covering a 10,000-fold concentration range. The effect of BRAF inhibition on cell viability was reported relative to vehicle-treated controls following 48-hours of treatment over the range of drug doses.

**Figure 1:**
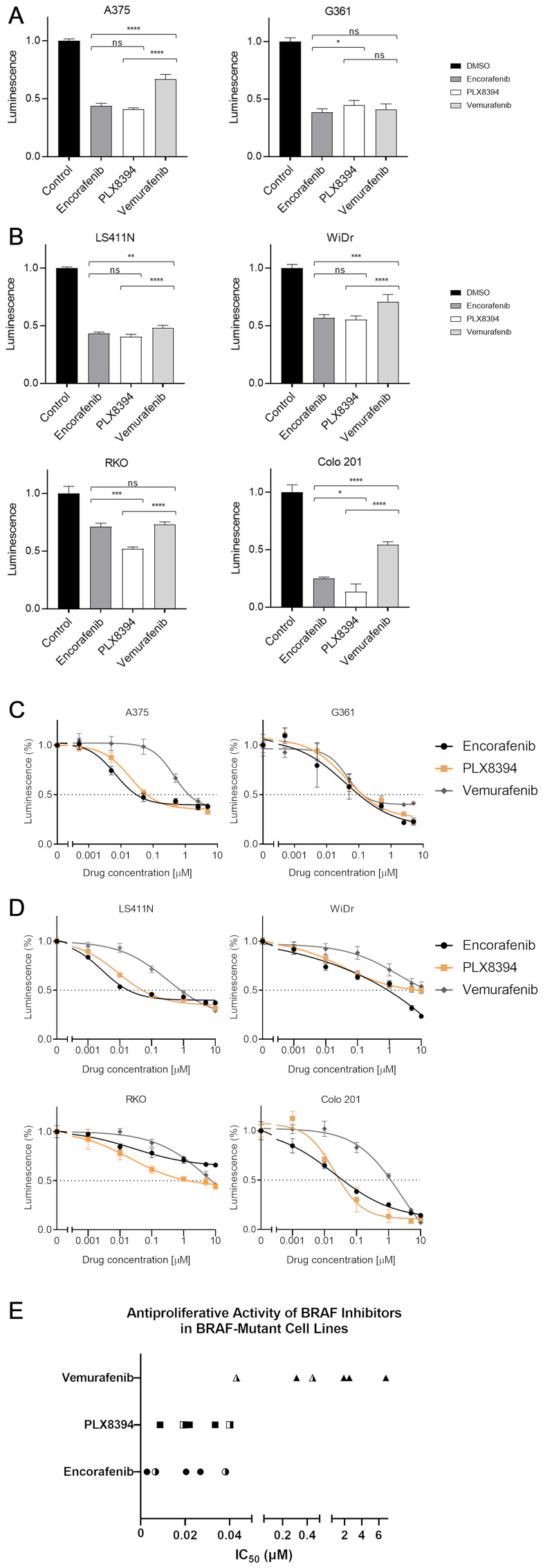
BRAF inhibitor-induced changes in cell viability. BRAF-mutant melanoma (**A**) and colorectal cancer (**B**) cell lines were treated with respective inhibitors at 1 μM for 48 hours prior to measuring luminescence. Changes in viability were measured using RealTime-Glo™ MT Cell Viability Assay and reported relative to vehicle-treated control. Mean values with standard deviations plotted from 5 replicates. Significance levels legend: Not significant (NS) *P* > 0.05; ^*^ *P* ≤ 0.05; ^**^ *P* ≤ 0.01; ^***^ *P* ≤ 0.001; ^****^ *P* ≤ 0.0001. N.B. Statistics for all drugs *vs* control not shown for ease of display and interpretation, all 3 compounds highly significant (*P* < 0.0001). Drug dose response curves. Sensitivity to respective BRAF inhibitors was evaluated in melanoma (**C**) and colorectal (**D**) cell lines. Cells were treated with 10,000-fold dilution series (0.001 μM to 10 μM) of respective BRAF inhibitors for 48 hours. Cell viability was assessed using RealTime-Glo™ MT Cell Viability Assay and reported relative to vehicle-treated control. The x-axis represents the log transformed inhibitor dose concentration. Mean values with standard deviation plotted from five replicates. (**E**) Derived IC_50_ values of BRAF inhibitors in melanoma and CRC cell lines (half-filled and filled symbols, respectively).

Comparison of a single dose potency (1 μM for all compounds) was first performed. All three drugs significantly inhibited cell viability compared with control (*P* < 0.0001, all cell lines, Figure 1). In melanoma cells, the reduction in relative viability was 56.5–61.7% following treatment with encorafenib, 55.4–59.2% with PLX8394 and 33.3–59.1% with vemurafenib (Figure 1A). A range of responses were observed in the colorectal cell lines. Colo 201 was relatively sensitive to BRAF inhibition (reduction in viability with encorafenib 74.9%, PLX8394 86.5% and vemurafenib 45.7%, respectively). RKO showed the smallest reduction in viability (encorafenib 29.0%, PLX8394 48.2% and vemurafenib 26.8%) (Figure 1B).

Encorafenib was significantly more effective than vemurafenib in 4 out of 6 cell lines (*P* = 0.0023 LS411N, *P* = 0.0002 WiDr, *P* < 0.0001 A375 and Colo 201). Similarly, PLX8394 showed greater potency than vemurafenib in 5 out of 6 cell lines (*P* < 0.0001 A375, LS411N, WiDr, RKO, and Colo 201, Figure 1) clearly demonstrating that both encorafenib and PLX8394 are more potent than vemurafenib. In half of the cell lines (A375, LS411N, and WiDr), there was no significant difference with respect to single dose potency between encorafenib and PLX8394 (*P* > 0.05). In the remaining lines, PLX8394 demonstrated greater efficacy in two lines compared to encorafenib (Colo 201 *P* = 0.016 and RKO *P* = 0.002), with encorafenib showing superiority in one line (G361 *P* = 0.0311) (Figure 1).

Cell viability readouts over the 0.001–10 μM dose range were used to construct dose response curves and calculate IC_50_ values. PLX8394 and encorafenib appeared highly comparable, with curves closely related in all lines except RKO (Figure 1C and 1D) and calculated IC_50_ values below 40 nM (Figure 1E). Interestingly, with both encorafenib and PLX8394, similar IC_50_ values were observed in both CRC and melanoma cells. By contrast, vemurafenib IC_50_ values were generally higher and the drug appeared an order of magnitude less effective in CRC (IC_50_ 0.3141–6.824 μM, mean value 2.897 μM) compared to melanoma (IC_50_ 0.0431–0.4441 μM, mean value 0.2436 μM) (Table 1).

**Table 1:** Half maximal inhibitory concentration of respective BRAF inhibitors

Cell Line	IC_50_ (μM)		
	Encorafenib	PLX8394	Vemurafenib
**Melanoma**			
A375	0.0066	0.0193	0.4441
G361	0.0382	0.0402	0.0431
**CRC**			
LS411N	0.0028	0.0087	0.3141
WiDr	—	0.0336	1.906
Colo 201	0.0205	0.0203	2.544
RKO	0.0269	0.0219	6.824

The anti-proliferative activity of Encorafenib, PLX8394 and vemurafenib was evaluated against CRC and melanoma lines expressing BRAF V600E. IC_50_ for encorafenib in WiDr was not determined.

Clinical studies have previously determined peak concentration achieved in serum (C_max_) following administration of varying doses (50–550 mg, once daily) of encorafenib. In the BEACON CRC trial, the encorafenib 300 mg once daily dosing was used, with a peak plasma concentration of 5.4 μM observed at this dose [2, 8]. This *in vitro* evaluation of encorafenib predicts its clinical activity at dose levels below this concentration, supporting the notion that encorafenib is likely to confer therapeutic benefit at clinically achievable concentrations (Figure 1C–1E). At the recommended phase 2 dose of PLX8394 (co-administered with cobicistat), the C_max_ is 55 μM, C_min_ 4 μM and C_avg_ 27 μM (personal communication, Plexxikon) thus well surpassing the concentrations required for maximal inhibitory effect *in vitro*. In summary, examination of the dose response curves and the IC_50_ values show high degrees of similarity between encorafenib and PLX8394 and which are clearly distinct from those obtained using vemurafenib.

Time-dependent pathway reactivation in BRAF mutant CRC has been proposed as underpinning its lack of single agent efficacy in this disease. The degree of pathway reactivation with encorafenib, PLX8394 and vemurafenib was compared by assessing levels of ERK phosphorylation across CRC and melanoma cell lines following continuous exposure to each agent (1 μM) or vehicle (0.2% DMSO). Cells were lysed following 3, 24, and 48-hour incubation with each BRAF inhibitor or DMSO.

**Figure 2:**
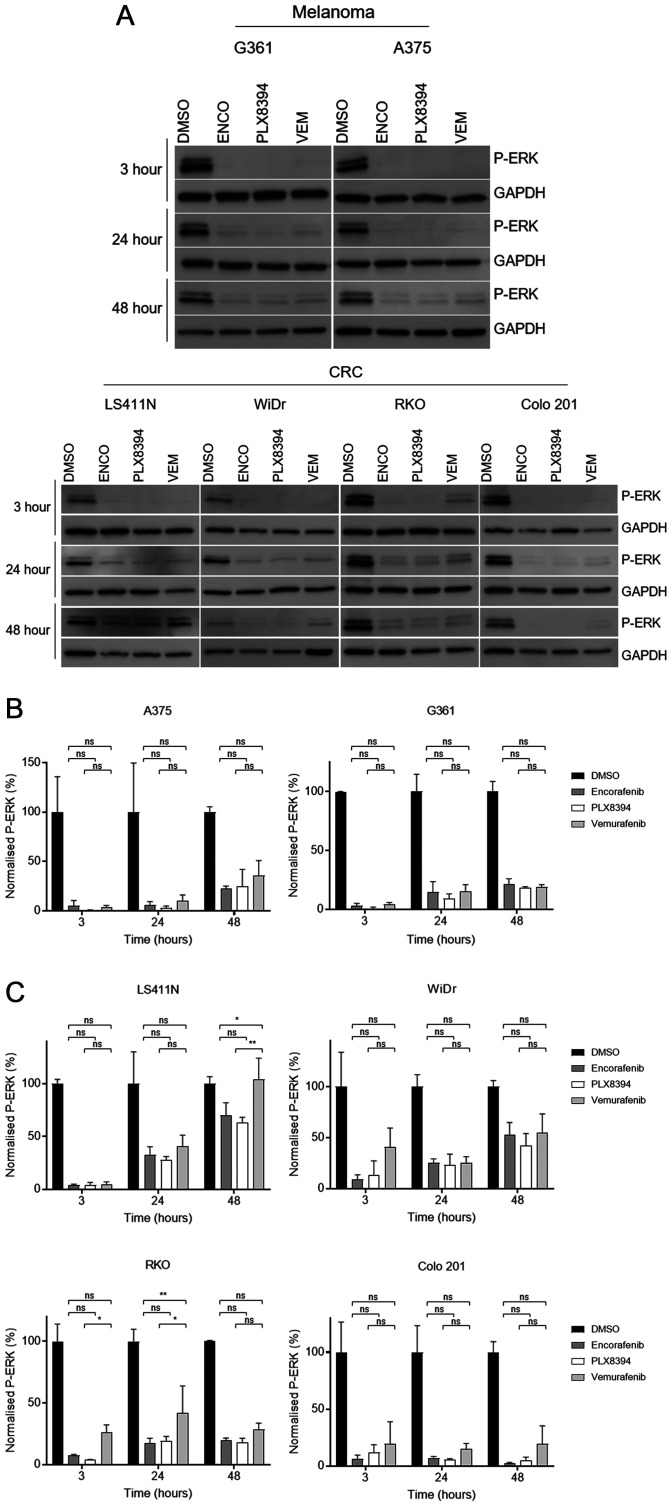
Assessment of MAPK pathway reactivation following BRAF monotherapy in BRAF-mutant cells. (**A**) The level of ERK phosphorylation (P-ERK) following treatment with vehicle (0.2% DMSO) or 1 μM of each drug was determined by western blot. Results for both melanoma (top) and CRC (bottom) cell lines shown at 3, 24, and 48 hour time points. GAPDH loading control for each blot shown. Experiments performed in triplicate with representative results shown. (**B**–**C**) Western blot quantification by densitometry. Phosphorylated ERK (P-ERK) levels quantified and normalised to loading control in melanoma and CRC-derived cells (B and C, respectively). Expressed as percentage of control for each time point. Mean values with standard deviation plotted (*n* = 3). Significance levels legend: NS *P* > 0.05; ^*^ *P* ≤ 0.05; ^**^ *P* ≤ 0.01; ^***^ *P* ≤ 0.001; ^****^ *P* ≤ 0.0001. N.B. Statistics for all drugs *vs* control not shown for ease of display and interpretation, all 3 compounds highly significant (*P* < 0.0001).

Both melanoma lines showed near-complete inhibition of P-ERK following 3-hour exposure to each drug, as previously reported [9, 10]. P-ERK levels began to recover as early as 24 hours post-treatment (Figure 2A). CRC lines also demonstrated a high degree of P-ERK suppression at the earliest time point, albeit not as complete as observed in melanoma (Figure 2A). After 24 hours there was evidence of partial reactivation of the pathway, showing similar durability of response as seen in the melanoma lines. As expected, the greatest reactivation of P-ERK was observed at 48 hours, demonstrating time-dependent reactivation. This was notably more evident in CRC lines, particularly LS411N and WiDr, with melanoma lines showing minimal change between 24 and 48 hours. Colo 201 appeared relatively sensitive to all inhibitors, a finding consistent with cell viability results from this line.

Comparing the extent of pathway reactivation of the different compounds, vemurafenib appeared the least effective at maintaining pathway inhibition (Figure 2A). Densitometry was performed on relative P-ERK levels after normalisation to loading control. At each time point, all three inhibitors significantly (*P* < 0.01 to *P* < 0.0001) suppressed levels of P-ERK compared to vehicle-treated controls. However, statistically significant differences between the different drugs were only observed at single time points in two CRC cell lines (RKO 24 hours and LS411N 48 hours). In these instances, both PLX8394 and encorafenib demonstrated superior suppression versus vemurafenib. Crucially, time dependent pathway reactivation was seen with PLX8394 and there was no significant difference with respect to pathway inhibition/reactivation between encorafenib and PLX8394 at any time point in any cell line (Figure 2B and 2C). Thus, despite the differing mechanisms and drug binding behaviour of these compounds, time dependent reactivation of P-ERK appears to be equivalent. Thus, encorafenib and PLX8384 have similar *in vitro* activity and downstream pathway inhibition/reactivation profiles in BRAF mutant CRC but possess greater potency than vemurafenib and in some cell lines less time-dependent pathway reactivation.

## DISCUSSION

Our results suggest that given the very similar dose response curves and pathway reactivation profiles between the BRAF inhibitors encorafenib and PLX8394, it will be interesting to evaluate PLX8394 as a potential substitute for encorafenib as part of a combinatorial approach with EGFR and MEK inhibition in patients with BRAF mutant CRC. Intuitively, given that pathway reactivation *via* RAF dimerization is thought to be an important mechanism limiting the efficacy of BRAF inhibitors in BRAF mutant CRC, it might be expected that the use of a paradox breaker inhibitor could provide enhanced pathway inhibition and hence enhanced efficacy. However, whilst PLX8394 was superior to vemurafenib, its unique mode of action did not translate to major differences when compared to another group 1 inhibitor encorafenib, a drug which is approved in combination with cetuximab for the treatment of patients with BRAF mutant CRC [2].

PLX8394 markedly reduces the levels of RAS-dependent full length BRAF-BRAF and BRAF-CRAF dimers [4]. Binding of PLX8394 to either BRAF protomer disrupts BRAF homodimerization but binding to CRAF is required to interfere with BRAF-CRAF dimer formation. In contrast the drug has no effect on CRAF homodimer formation or on ARAF-containing dimers. PLX8394 activates CRAF homodimers *via* negative co-operativity, transactivating the unbound CRAF protomer and activating ERK signalling. ERK signalling was induced by PLX8394 in cells engineered to overexpress CRAF and in cells with BRAF knocked out, both conditions enhancing the formation of CRAF homodimers. Thus, PLX8394 fails to inhibit CRAF homodimerization and activates CRAF homodimers. Tumours in which MEK-ERK signalling is driven by RTK activation or mutant RAS express CRAF homodimers. Mutant NRAS causes the formation of all RAF dimers including CRAF homodimers and as predicted PLX8394 was ineffective in an NRAS mutant mouse model. The central importance of CRAF in mediating ERK pathway activation in RAS mutant or RAS/RAF wild-type cells treated with BRAF inhibitors was shown in earlier studies in which P-MEK and P-ERK induction could be reversed with CRAF knockdown but not with BRAF knockdown [5, 6].

Thus, whilst PLX8394 is highly effective against BRAF V600E monomers and dimers it will not be active in situations where RAS is activated. RAS activation (caused by EGFR-mediated activation) is central to pathway reactivation in BRAF mutant CRC treated with BRAF inhibitors [3]. Any resulting CRAF homodimers driven by activated RAS will themselves be activated by PLX8394, thus causing pathway reactivation, negating any potential benefit over group 1 inhibitors. Indeed, the disruption of BRAF-CRAF dimerization by PLX8394 will enhance the formation of CRAF homodimers by increasing the amounts of free CRAF protomers. Thus, the lack of difference in pathway reactivation comparing the highly potent group 1 BRAF inhibitor encorafenib with the paradox breaker PLX8394 in treated BRAF mutant cells is unsurprising.

The stark difference in the clinical activity of vemurafenib between BRAF mutant melanoma and BRAF mutant CRC was attributed to the complete lack of pathway reactivation in the former, principally related to absent or low level EGFR expression [3]. Although less marked than in CRC cell lines we did find evidence of time-dependent pathway reactivation after total shut-down at 3 hours in the melanoma cell lines. Neil Rosen’s group also clearly demonstrated that vemurafenib treatment of BRAF mutant melanoma cells caused receptor tyrosine kinase-mediated (RTK) activation of RAS *via* Spry2 downregulation, with CRAF activation and pERK rebound after initial profound inhibition [9]. This pathway reactivation was dependent on CRAF-containing dimers and was sensitive to combined MEK inhibition. Thus, similar to BRAF mutant CRC, the efficacy of BRAF inhibitors in melanoma was limited by the relief of feedback inhibition of RTK-RAS-RAF with consequent MEK-ERK pathway reactivation, an effect that could be blocked with combined BRAF/MEK inhibition. Whilst this strategy is highly effective in melanoma [11], the impact in CRC is minimal [12]. Enhanced pathway reactivation in CRC relative to melanoma might be suggested as accounting in part for the differences clinically, however combined BRAF/MEK blockade is also highly effective in BRAF mutant lung cancer [13], a disease which like CRC is characteristically an EGFR expressing cancer [14].

Whilst we are not aware of any data that interrogates pathway reactivation upon BRAF inhibition in BRAF mutant lung cancer models, pathway reactivation after KRAS G12C inhibition in G12C mutant lung cancer has been analysed [15]. There is an identical RTK-driven activation of wild-type RAS (not G12C) that reactivates MEK-ERK signalling after initial pathway shutdown and which can be inhibited by vertical pathway suppression. This reactivation is seen in both G12C lung cancer and CRC cell lines to an equivalent degree. However, despite comparable levels of signalling reactivation, the impressive responses to G12C monotherapy described in lung cancer have not been observed in G12C mutant CRC, a parallel to the disparities in response to BRAF inhibition in CRC [16]. These data suggest that other reasons beyond pathway reactivation might limit the efficacy of BRAF inhibition in BRAF mutant CRC. We have recently reported that response rate, progression free survival and overall survival is significantly greater in BRAF mutant CRC patients with the BM1 transcriptional sub-type than the BM2 sub-type when treated with combined BRAF/MEK/EGFR inhibition [17]. BM1 is immunologically enriched compared with BM2 and this is likely to be pertinent to this differential efficacy [18, 19]. Transcriptional and immunological context has also been confirmed as a key determinant of efficacy for BRAF inhibition in BRAF mutant melanoma [20, 21]. Whilst rational vertical pathway combination therapy based on dissection of the mechanisms of pathway reactivation has been pivotal to improving upon the results with BRAF inhibitors, the differential efficacy of BRAF inhibition across different BRAF mutant cancers is more likely to be related to the contextual differences, particularly the immune context of the BRAF mutation, rather than differences in the degree of pathway reactivation. Intriguingly, given the differences in clinical outcome with G12C blockade in CRC and lung cancer, long term cures in G12C models were dependent upon immune system engagement [22].

One of the limitations of this study is that we only analysed the impact of the drugs used as single agents and did not analyse their impact in combination with MEK inhibitors. We were specifically interested in directly comparing PLX8394 with encorafenib as a single agent to see whether it was either equivalent or superior to encorafenib thus supporting its clinical investigation in CRC. Importantly, updated outcome data from the BEACON trial presented at ASCO 2020 show the improvement in progression free survival and overall survival over control is virtually identical with the triplet containing the MEK inhibitor binimetinib as it is with the cetuximab/encorafenib doublet not containing MEK inhibition. As a result, the EMA have only approved the doublet in BRAF mutant CRC and not the triplet, the FDA have similarly approved the doublet regime alone and the further development of the combination in the first line setting will be as the doublet without MEK inhibition [23].

In summary, we have shown that the strategy of using a paradox breaker BRAF inhibitor such as PLX8394 in order to reduce pathway reactivation through reduced RAF dimerization in BRAF mutant CRC is as effective as optimal group 1 inhibitors such as encorafenib. The degree of pathway reactivation is similar likely due to the inability of PLX8394 to inhibit CRAF homodimer formation and the activation by PLX8394 of such dimers. As with the continued clinical development of BRAF inhibition in BRAF mutant cancers, the activity of PLX8394 should be investigated as part of a combination with other drugs that limit pathway reactivation such as MEK and EGFR inhibitors. However, the general strategy of targeting RAF dimerization in RAS/RAF mutant colorectal cancers is unlikely to qualitatively transform the outcomes with targeted therapies without appropriate attention to the unique biology of BRAF and KRAS mutant CRC [17, 24, 25]. The lack of confirmed objective responses in BRAF mutant CRC, in contradistinction to melanoma, to the RAF dimer inhibitor lifirafenib which inhibits all RAF isoforms as well as EGFR and KRAS supports this hypothesis [26].

## MATERIALS AND METHODS

### Cell culture

All cell lines were purchased from ATCC (Manassas, VA, USA) and certified authentic and contamination free on arrival. Cells were grown in the following media supplemented with fetal bovine serum (FBS): DMEM (RKO, WiDr, A375), McCoy’s 5a (G361) and RPMI-1640 (LS411N, Colo 201) in humidified incubators at 37°C with supplemental 5% CO_2_. Cells were regularly tested for contamination with mycoplasma with EZ-PCR Mycoplasma Test Kit (Biological Industries, Israel).

### Drug assessment and determination of cell viability

Encorafenib was obtained from Cambridge BioScience (UK) and vemurafenib from Stratech Scientific Limited (UK). PLX8394 was kindly supplied by Plexxikon Inc (Berkeley, CA, USA). Compounds were diluted in dimethyl sulfoxide (DMSO) covering a 10,000-fold concentration range. Low passage cells (less than twenty passages) were used for experiments, with regular resuscitation of early passage stock to maintain line fidelity.

Cell viability was assessed using the RealTime-Glo™ MT Cell Viability Assay (Promega, USA). Optimal seeding densities were determined for each of the lines to ensure assay linearity. Experiments were performed in reduced serum media (5% FBS) with the addition of drug for 48 hours prior to determination of cell viability. At the end point, luminescence was measured using a microplate reader (EnSpire, PerkinElmer). Readings were taken for five replicates for each drug condition. Drug response curves were generated and accompanying IC_50_ values calculated in GraphPad Prism version 8.3.0, GraphPad Software (San Diego, CA, USA). Luminescence values for 1 μM drug concentration were reported relative to vehicle treated controls (0.2% DMSO) and compared using two-way ANOVA with Tukey’s post-test.

### Western blotting

Cells were plated at reduced FBS concentration and were exposed to inhibitors at 1 μM or 0.2% DMSO. Lysates were quantified and 20 μg total protein loaded onto 10–12% Mini-PROTEAN TXG Precast Gels (Bio-Rad, USA). Primary antibodies were obtained from Cell Signalling Technology (USA) and the following clones and dilutions were used: P-ERK [Phospho-p44/42 MAPK (Erk1/2) (Thr202/Tyr204) #9101, 1:1000] and GAPDH [GAPDH (D16H11) XP (R) #5174, 1:1000]. The P-ERK antibody has been previously cited for studying downstream effects of BRAF inhibitors on MAPK pathway activity [3], and in-house validation of dilutions was performed. Blots were probed with appropriate secondary HRP-conjugated antibodies and detected using chemiluminescence on a Fusion FX6XT digital imaging system (Vilber Lourmat, Germany).

Densitometry was performed for P-ERK and GAPDH for each cell line at each time point. All experiments were conducted in triplicate. Two-way ANOVA with Tukey’s post-test was performed on densitometry values, again in GraphPad Prism Software.

## Abbreviations

CRC: colorectal cancer
MAPK: Mitogen-activated protein kinase
EGFR: epidermal growth factor receptor
(P)-ERK: (phosphorylated) extracellular signal-regulated kinase
ATP: adenosine triphosphate
IC_50_: half maximal inhibitory concentration
C_max/min/avg_: maximum/minimum/average blood plasma concentration
DMSO: dimethyl sulfoxide
RTK: receptor tyrosine kinase
BM1/2: BRAF mutant subtype 1/2
DMEM: Dulbecco’s Modified Eagle’s medium
RPMI-1640: Roswell Park Memorial Institute media
FBS: Fetal bovine serum
